# Size is relative: use of relational concepts by wild hummingbirds

**DOI:** 10.1098/rspb.2021.2508

**Published:** 2022-03-30

**Authors:** Theo Brown, T. Andrew Hurly, Susan D. Healy, Maria C. Tello-Ramos

**Affiliations:** ^1^ School of Biology, University of St Andrews, St Andrews KY16 9TH, UK; ^2^ Department of Biological Sciences, University of Lethbridge, Lethbridge, Alberta, Canada

**Keywords:** foraging, hummingbirds, relational concepts, *Selasphorus rufus*, transposition

## Abstract

Rufous hummingbirds (*Selasphorus rufus*) will readily learn the location and the colour of rewarded flowers within their territory. But if these birds could apply a relational concept such as ‘the larger flowers have more nectar’, they could forego learning the locations of hundreds of individual flowers. Here, we investigated whether wild male territorial rufous hummingbirds might use ‘larger than’ and ‘smaller than’ relational rules and apply them to flowers of different sizes. Subjects were trained to feed consistently from one of two flowers. Although the flowers differed only in size, the reward was always contained in the same-size flower. The birds were then tested on a choice of two empty flowers: one of the familiar size and the other a novel size. Hummingbirds applied relational rules by choosing the flower that was of the correct relational size rather than visiting the flower of the size rewarded during training. The choices made by the hummingbirds were not consistent with alternative mechanisms such as peak shift or associative learning. We suggest that while hummingbirds are very good at remembering the spatial locations of rewarding flowers, they would be able to use relative rules when foraging in new and changing environments.

## 1. Introduction

‘Larger’ is a relational concept, an abstract relationship between two things. Relational concepts such as ‘larger than’ or ‘smaller than’ are independent of specific objects, and as such, these rules can be transferred (or ‘transposed’) to different objects at different times since the important information is in the rule itself rather than the specific items [1]. In this way, a single object can be both larger and smaller than other objects depending on the comparison being made. To test the ability to form a relational rule between two objects, typically a subject is presented with a single pair of stimuli that differ in a single dimension (e.g. size, where one stimulus is larger than the other), one of which is rewarded and one that is not (e.g. stimulus medium is rewarded and stimulus small is not). At testing, the subject is presented with a pair of stimuli containing one of the previously presented stimuli and a novel stimulus of the same kind but of differing size (e.g. stimulus medium versus stimulus large). In this example, choosing the novel rather than the previously reinforced stimulus suggests that the subject has used the relational rule between the objects rather than the absolute size of the rewarded item (i.e. relational rule = the larger of two items is rewarded).

Being able to form and use abstract relationships between objects has been considered a cornerstone of human cognition [2], an ability that develops through infancy as human children acquire language [3,4]. Although in children and adults, the ability to use relational concepts is thought to be the basis of abstract thinking, a growing number of other species, vertebrates and invertebrates, appear to use relational concepts. These species include chickens (*Gallus gallus domesticus*) [5], pigeons (*Columba livia*) [6], black-billed magpies (*Pica hudsonia*) [7], African grey parrots (*Psittacus erithacus*) [8], sea lions (*Zalophus californianus*) [9], horses (*Equus caballus*) [10], dolphins (*Tursiops truncates*) [11], honeybees (*Apis mellifer*) [12,13] and tufted capuchin monkeys (*Cebus apella*) [14].

The question of whether and when animals use relational rules has, however, been mostly studied in the laboratory or in captive situations where the animals undergo intensive training. Here we examine whether wild, free-living hummingbirds might also use relational rules in an ecologically relevant scenario.

During the boreal summer, male rufous hummingbirds (*Selasphorus rufus*) hold territories containing hundreds to thousands of flowers. These birds will adjust the size of the territory they defend in relation to both the density of flowers [15,16] and the flower species within the territory [17]. When discriminating between patches of flowers, these birds appear to also be able to use an approximate number system [18] and can reliably discriminate between the quality of a flower's reward both in nectar concentration and volume [19]. The ability to discriminate between the quality of two patches, however, has only been observed after hummingbirds have experienced the contents of the flowers. Given that the flowers hummingbirds forage from vary greatly in the amount and quality of the resource they offer [20,21] here we asked whether rather than learning the exact value of each individual flower, territorial hummingbirds might use relational rules when choosing which territories to defend or which flowers to prioritize. Given that larger flowers typically offer more nectar [22], hummingbirds might apply relational rules such as preferring the larger of two flowers or a meadow containing more larger flowers over a meadow with more smaller flowers.

To test this possibility, we first trained hummingbirds to associate either the larger or smaller of two flowers with a reward, then we tested whether the hummingbirds would transfer the conceptual rule to flowers of different sizes by ‘upscaling’ or ‘downscaling’ the flowers offered. During these transfer tests, if hummingbirds use *relational rules* when foraging, we expected birds to visit the flower of the appropriate relative size rather than the flower of the absolute size it had experienced in training. There are two alternative possibilities: (i) if the hummingbirds displayed a directional preference for the flower closer in size to the flower that was rewarded during training, this would be a response consistent with *peak shift*; (2) if the birds visited the flower of the same absolute size as the rewarded flower during training whenever it was available during the tests, their behaviour would be congruent with *associative learning*.

## 2. Methods

### (a) Field site and subjects

We tested the use of relational concepts when foraging in 16 free-living, male rufous hummingbirds that were defending individual territories at Westcastle Valley, Alberta, Canada in the eastern Rocky Mountains (49°349153′ N, 114°410864′ W) during the summer months of 2019. In order to reliably identify each individual hummingbird, we first identified all hummingbirds within the valley that were defending a territory around an artificial feeder that contained 16% sucrose solution. We then marked each hummingbird with a non-toxic marker (Jiffy Eco-marker Ink) both beneath the gorget and on the back of the bird. One day after a bird had been marked, we trained him to feed from an artificial flower. The artificial training flower (hereafter referred to as ‘flower’) was a thin foam circle with a diameter of 6 cm attached to the top of a 60 cm wooden stick. Inserted into the centre of the foam circle was a syringe cap with a capacity of 600 µl. This syringe cap sat in a centrifuge tube that was taped to the top of the wooden stick. Birds were trained to feed from a single yellow flower containing 25% sucrose. Once the bird was feeding from the training flower circa every 10 min, the flower was moved in sequentially larger increments. Moving the flower prevented the birds from associating the spatial location with the reward since, for these birds, the spatial location of a flower is a salient cue [23–25]. After each visit from the hummingbird, the flower was moved and refilled. This process was repeated until the hummingbird consistently followed moves of 100–150 cm, a process that took about an hour. We then started the experimental training.

### (b) Experimental protocol

**Figure 1.**
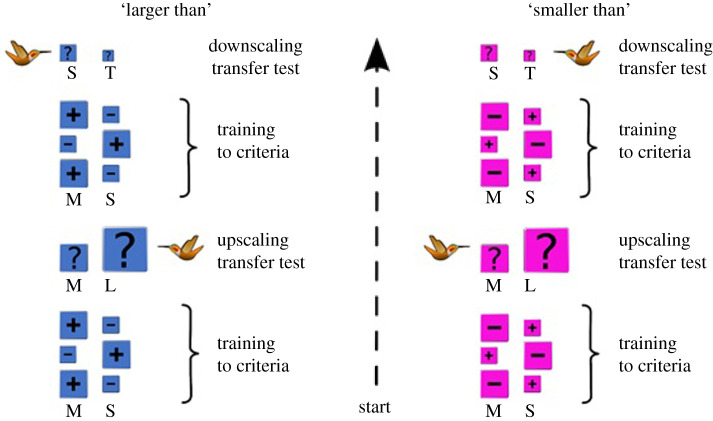
Schematic of the experimental treatments. Birds were presented with two flowers. Depending on the treatment, either the medium or the small flower would contain 120 µl of 25% sucrose solution (+ signals the rewarded flower), the other flower containing 5% sucrose (− signals the low reward flower). During training, birds had to visit both flowers at least once and visit the rewarded flower first (+) three times in a row to reach both criteria. During the upscaling transfer test, the birds were presented with a medium and a large flower. A second reinforcement training session was followed by a downscaling transfer test where a small and a tiny flower were presented. The order and colour of flowers in each treatment were counterbalanced across birds. Flower size: tiny = 4 cm^2^, small = 9 cm^2^, medium = 25 cm^2^, and large = 64 cm^2^. Training of the two treatments was counterbalanced between the birds. The arrow shows the order of training and tests. Illustration credit: Eduardo Tello-Ramos. (Online version in colour.)

Individual birds were presented with two square flowers, one ‘medium flower’ (25 cm^2^) and one ‘small flower’ (9 cm^2^) separated by 15 cm (figure 1). The experiment was divided into two treatments, *larger than* and *smaller than*. In the *larger than* treatment, the medium flower contained 120 µl of 25% sucrose solution while the small flower contained 120 µl of the much less preferred 5% sucrose solution [26]. We manipulated sugar concentration because territorial male rufous prefer small but frequent meals, filling their crops to a 10% capacity as they benefit from carrying less weight when chasing off an intruder or displaying to a female [27]. For territorial males, then, a flower with a higher sucrose concentration would be more desirable than a flower with a higher volume of sucrose but lower sugar concentration. Within a bout, birds would first visit the array and sample both flowers. After each bout, both flowers were moved 50–100 cm and placed in a different orientation in relation to each other in a pseudo-random order. This was done to prevent the birds from using the spatial location or relative orientation of the rewarded flower when identifying the reward [28]. Between bouts, the artificial flowers were exchanged for new flowers of the same colour and size to prevent the birds from associating any specific mark or artefact of the flower with the reward [29]. This procedure was repeated until two criteria had been reached. First, the bird had sampled both the small and medium flowers at least once. Second, the bird had visited the rewarded flower, the medium flower in this case, first in each of three consecutive foraging bouts. Reaching this second criterion showed that the bird had learnt that the larger flower of the two held the higher 25% sucrose solution.

After the second criterion was met, the bird was presented with a probe test (either upscaling or downscaling). In the upscaling test, the bird was presented with a medium and a ‘large’ flower (64 cm^2^), both unrewarded. If the hummingbird was using a relational concept to identify the rewarded flower (the larger of two flowers), then the bird should visit first the large flower even though that flower was not the absolute size of the flower that had been rewarded during training. The bird was allowed to visit the test array once. Immediately after this transfer test, the bird was presented again with a training array (medium and small flowers), with the medium flower rewarded with 25% sucrose solution and the small flower with 5% sucrose solution. Again, the bird had to reach both criteria to ensure that it had associated the medium flower with the high reward. A second test involved downscaling the array, wherein the bird had to choose between a small flower and a ‘tiny flower’ (4 cm^2^). If the bird was using relational concepts, he should visit the small rather than the tiny flower even though that flower was not rewarded during training (figure 1).

In training for the s*maller than* treatment, the birds were again presented with a medium and a small flower, except that during this treatment it was the small flower that contained the higher 25% sucrose solution. Again, the birds had to reach the two criteria before they were presented with an upscaling transfer test. If the birds used a relational rule, when presented with a medium and a large flower, they should visit the medium flower since it was the smaller of the two options. After a second training session where the birds had fed from the small rather than the medium flower, we presented them with a downscaling transfer test. In this test, we presented a small flower and a tiny flower. If the birds used a relational concept, then they should visit the tiny flower.

All birds completed the four transfer tests: medium upscaling, medium downscaling, small upscaling and small downscaling (figure 1). The colour (pink or blue) of the flowers in the *larger than* and the *smaller than* treatments were the same within a treatment but different between (figure 1). The order of the treatments and the colour of the flowers for each treatment were counterbalanced across the 16 birds. The order that the birds visited the two flowers in each bout was recorded throughout the training sessions and during the transfer tests. If for any reason the experiment was stopped during training (due to the end of the day or bad weather), the feeder was replaced during the inter-experimental period and when the experiment was resumed, the bird would have to reach both criteria again.

### (c) Statistical analyses

First, to determine whether the birds responded differently to the two training flower sizes (medium or the small flower rewarded), we used a repeated measures two-factor ANOVA with *birdID* as a random variable, *size* of the training flower and *order* of the training treatments as the two independent variables while also including the interaction between these two (using the function lme from the nlme package; [30]). The significance of model terms was tested using the Wald chi-square statistic.

We used a chi-square goodness of fit test to determine if there were differences in the total number of times birds choose the relative flower versus the non-relative flower during the four transfer tests.

To determine how the size of the training flowers, the direction or order of the tests affected the flower chosen during the four different tests, we used a general estimate equations (GEE) analysis. We included *birdID* as the grouping variable to control for repeated measures with an exchangeable correlation structure and a binary distribution for the dependent variable (using the function geeglm function from the geepack for R; [31]). We report the significance of model terms using the Wald chi-square statistic.

In order to determine whether individual birds repeated their choice for one of the flowers across the four tests, we calculated the proportion of the variation between and within individual birds, also known as intraclass correlation coefficient, using a repeatability test. The significance of individual consistency (at *α* = 0.05) was calculated with individual bird as a grouping variable, a binary distribution and 1500 permutations (rpt function in the rptR R package; [32]).

We compared the observed number of choices to the relative flower during the four transfer tests to what would have been expected under three different learning scenarios. (i) If birds had learned the relational size of the flowers, then birds should visit the relative-size flower (the large flower during the medium upscaling test, the small flower during the medium downscaling test, the medium flower during the small upscaling test and the tiny flower during the small downscaling test). (ii) If instead, birds had generalized a positive stimulus across a size gradient (i.e. peak shift), then birds were expected to visit the relative flower during the medium upscaling and small downscaling transfer tests but to show no preference during the medium downscaling and small upscaling tests. (iii) If hummingbirds had associated the exact size of a flower with the reward, then birds were expected to visit the absolute size flower rather than the relative flower during the medium upscaling and the small downscaling tests but during the other two tests, the birds would visit the two flowers at chance. We compared the observed visits for each scenario with the predicted visits using three multinomial goodness of fit tests with full enumeration (using the xmulti function from the xnomial R package; [33]).

## 3. Results

**Figure 2.**
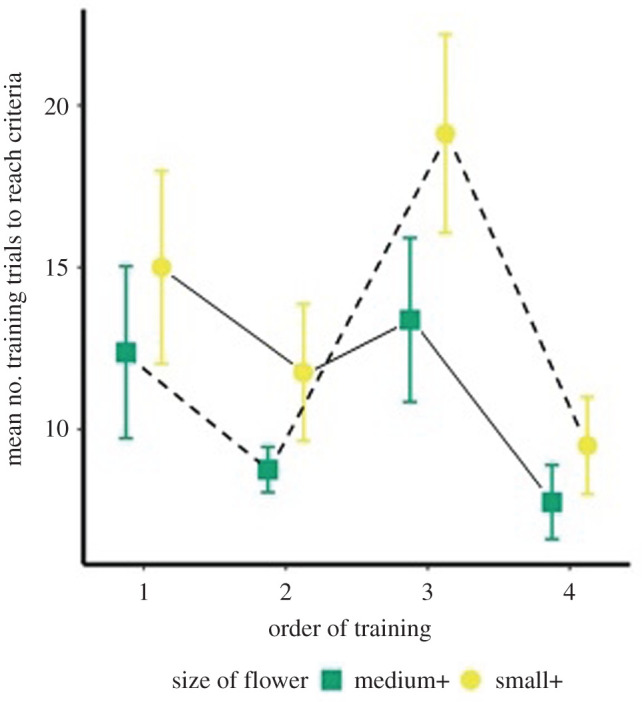
The mean (± s.e.) number of trials hummingbirds took to reach the criteria when the medium flower (green square) or the small flower (yellow circle) were rewarded and the effect of the training order (dash or solid lines). Half of the hummingbirds experienced medium-medium small-small (dash line) as the training protocol and the other half experienced small-small-medium-medium (solid line). (Online version in colour.)

All hummingbirds learned which of the two differently sized flowers was rewarded based on their size alone as all birds reached the criteria (mean no. of visits 12.2 ± 0.86 s.e.) across both trial types of the experiment. Birds took significantly more trials to learn that the smaller of two flowers was rewarded than they took to learn that the medium flower was rewarded (medium flower rewarded *µ* = 10.56 ± 1.02; small flower rewarded *µ* = 13.84 ± 1.35; $\chi_{1}^{2}=4.91$, *p* = 0.02; figure 2). No other comparisons were significant (electronic supplementary material).

**Figure 3.**
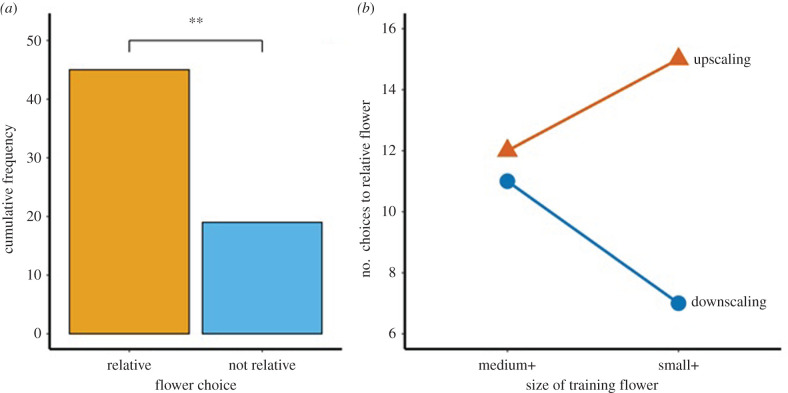
(*a*) Number of visits to the relative and not relative flowers during the probe upscaling and downscaling tests. *N* = 64 visits by 16 birds. **p = 0.001 as tested by a chi-square goodness of fit test. (*b*) Number of visits to the relative flower during the four transfer tests completed by 16 birds. Medium upscaling (large flower = relative), medium downscaling (small flower = relative), small upscaling (medium flower = relative) and small downscaling (tiny flower = relative). (Online version in colour.)

During the four transfer tests (two upscaling and two downscaling), the 16 birds chose the relative flower significantly more often than they chose the alternative flower (70.3% versus 29.7%; $\chi_{1}^{2}=10.56$, *p* = 0.001, *N* = 64; figure 3*a*).

Whether the birds visited the relative flower or not differed significantly among the four transfer tests. Birds visited more the relative flower during the upscaling tests (main effect of direction: $\chi_{1}^{2}=5.98$, *p* = 0.01), and this effect was more noticeable when the training flower had been the small flower (figure 3*b*). This interaction, however, was only borderline significant (interaction size:direction: $\chi_{1}^{2}=3.48$, *p* = 0.06). Whether the training flower had been a small or a medium flower had no effect on the number of visits to relative flowers during the four tests (main effect of size: $\chi_{1}^{2}=0.07$, *p* = 0.78). Fewer birds chose the relative flower as they completed more tests (main effect of order: $\chi_{1}^{2}=5.33$, *p* = 0.02, electronic supplementary material).

Frequencies of choices to the relational flower were compared to frequencies of three expected mechanisms. First, if birds were to use relational rules then birds were expected to visit the relational flower during each of the four tests. Second, if birds were following a size gradient (i.e. peak shift), then birds were expected to visit the flower that was more extreme relative to the size of the rewarded flower but to choose at chance (8/16) when the test did not include flowers similar to the size trained (e.g. if a bird was trained to M +, then in a choice between M versus L the bird would choose L but in a choice between S versus T the bird was expected to choose at chance). Third, if birds had associated the exact size of the rewarded flower, then birds were expected to choose the absolute flower whenever it was present at the tests and to choose at random when it was not present (table 1).

**Table 1.** The number of total choices expected under three different mechanisms and those observed to the relational flower during the four transfer tests.

	medium upscaling	medium downscaling	small upscaling	small downscaling
observed	12	11	15	7
relational	16	16	16	16
peak shift	16	8	8	16
associative^a^	0	8	8	0

^a^Because goodness of fit tests cannot be performed when a cell has the value zero, the value of 1 was added to each cell for observed and associative expected comparisons during the analysis.

There was no difference between the results observed and those predicted for the use of relational rules by the birds ($\chi_{3}^{2}=2.91$, *p* = 0.43, *N* = 64, table 1). There was, however, a significant difference between the observed and predicted choices if birds had generalized a positive stimulus across a size gradient (multinomial test: $\chi_{3}^{2}=14$, *p* < 0.01, *N* = 64, table 1). Birds mostly visited the relative flower even in the cases when a peak shift mechanism predicted visits to the relative flower at chance. The predictions for associative learning also differed significantly from the observed choices (multinomial test: $\chi_{3}^{2}=64.24$, *p* < 0.001, *N* = 64, table 1). Instead of visiting the absolute flower whenever it was present during the tests, birds mostly visited the relative flower during the medium upscale test (the large flower rather than the medium flower) and during the small downscaling tests the birds visited the two flowers at random.

Although there was variability in choices within and between individuals (electronic supplementary material), there was no evidence of systematic structure within this variation (*R* < 0.01, 95% CI = 0–0.2, *p* = 0.5).

## 4. Discussion

Hummingbirds readily learned which of two flowers, differing only in size, was rewarded (mean of 12 trials). Birds learned to visit the rewarded flower even though the location and position of the two flowers were moved between each visit. In general, with more experience with training protocol, the hummingbirds took fewer trials to reach the criteria (figure 2). However, when the hummingbirds had to reverse learn the association between the previously unrewarded flower, birds took more trials to learn the new association (figure 2, training order 3). This effect might be explained by proactive interference. Alternatively, as all three training sets that took hummingbirds the most trials to reach criteria went over 2 days (an afternoon and the next morning), it might simply be an effect of the interruption of the training protocol at the end of the day. Nonetheless, and compared to laboratory protocols, hummingbirds learned which of two flowers was rewarded readily.

Test results indicate a distinct (70%) preference for flowers of the relative size. The pattern of choices to the relational flower were consistent with those expected if hummingbirds used relational information to make their flower choice. The birds' choices were not, however, consistent with those they would have made if birds had employed peak shift to make their choice. For the birds' choices to have been consistent with peak shift, the birds would have chosen the flower closest in size to that of the trained flower while avoiding flowers closer in size to that of the flower that was unrewarded during training. They did not do this. A third possibility, associative learning, is that the birds could have visited the flower that was the same size as that of the flower rewarded during training or the one closest in size. Their choices were also not consistent with this explanation (table 1).

Evidence for the use of relative over absolute size was particularly strong from two of the test types (medium downscale, small upscale) in which the birds chose the flower of the same size as that of the previously unrewarded stimulus. Furthermore, in the medium upscale test, when the previously rewarded stimulus was present as the non-relational flower, birds did not choose the flower of this size.

There are numerous examples of animals learning to use relational rules following intensive training in the laboratory. Lazareva and colleagues determined that relational learning in pigeons is enhanced when training is done with multiple pairs of exemplars and when testing pairs are non-adjacent within the dimension being tested [34,35]. In the laboratory, for example, black-billed magpies that were trained for 100 trials using 64 exemplar pairs (e.g. 50 same ■ versus ■; 50 different ▲ versus ○) and then tested with 10 new transfer pairs (e.g. ▼ versus ◊), learned to use the relational concepts of ‘same’ and ‘different’ [7]. Free-flying bees too, which were trained for 80 trials to discriminate among six different exemplar pairs and were then tested with two novel transfer pairs, learned to use a relational rule between flowers of different sizes [13].

In this study, we did not specifically train hummingbirds to use a relational rule by presenting birds with multiple exemplars and rewarding only those visits that followed a relational rule. Rather, we presented a single exemplar in the form of a pair of flowers of different sizes and asked whether the birds remembered the rewarded flower by its absolute size or by its size relative to the size of the unrewarded flower. Tests, following a mean of 12 training trials, suggest that birds employed relative information when making their first choice in a novel comparison of flower size. This does not mean that they did not remember the absolute size, but that on average, their first tendency was to use relative-size information.

Hummingbirds use relational assessments in other contexts as well. In a spatial context for example, Henderson *et al*. [24] trained hummingbirds to forage from pairs of identical flowers presented at different heights from the ground (50 versus 70 cm, or 70 versus 90 cm) with only the 70 cm flower rewarded. When trained with one pair and then tested with the other, birds showed evidence of having remembered the rewarded flower in terms of its height relative to the height of the non-rewarded flower, not in terms of its absolute height [24]. A similar effect was found for the horizontal plane when hummingbirds learned to visit a central flower within a five flower cross that was moved for a test probe [36]. The use of the relational rule (i.e. centre flower is rewarded) was only found when the flowers were close together (less than or equal to 40 cm). When flowers were placed further apart hummingbirds visited the flower at the absolute spatial location. Finally, hummingbirds can employ numerical ordinality to recognize which flower in a linear array is rewarded [37], a different type of use of relational information.

Why do hummingbirds so readily use relative information when recalling flower size, height and small-scale position? To sustain a positive energy balance, hummingbirds visit hundreds of flowers each day [16,38]. While numerous studies show that hummingbirds primarily use spatial location when returning to feed from a single rewarded flower (e.g. [23,39]) is not yet clear that hummingbirds remember the location of each and every one of the flowers within their territory. Instead, hummingbirds might use a combination of different types of information depending on which information is more relevant at the time (e.g. time information: [40,41], or the colour of a flower: [42]). The results from these studies combined with the present results suggest that learning and using relational information that can be applied to different foraging contexts might be part of a hummingbird's ‘foraging toolbox’.

There might be, however, some limitations to the application of relational rules in the field. During the small downscale tests, for example, the birds' choices were no different from chance. Why the birds behaved differently in this condition is not clear but their choices were not consistent with peak shift or associative condition as instead of choosing the flower of the absolute or closest size to the size of the rewarded flower during training, birds chose at random. The lack of clear preference between absolute and relative size observed in the small downsize tests where the relational flower was tiny could be influenced by numerous factors. Large flowers may naturally be preferred over small flowers because they tend to contain more nectar in nature [22]. There is a hint of such a relationship in the learning data in that it required more trials for birds to learn that the small flower was rewarded than that the medium flower was rewarded (figure 2). Such natural preferences could also interact with difficulty in size discrimination in that smaller differences seem more difficult to assess than larger differences [43]. The difference between the large and medium flowers was 39 cm^2^, whereas the difference between the small and tiny flowers was only 5 cm^2^. The ratios of the size differences preserve this inequality, although seemingly less extreme (2.65 versus 2.25). During the medium downscaling tests, however, 11 of the 16 birds visited the small flower rather than the tiny flower, even though the small flower had been unrewarded during training. This suggests that hummingbirds could perceive the difference between the two flowers. However, to rule out the possibility that small size differences are more difficult to detect or discriminate, future experiments should include training hummingbirds to favour one size flower over the other one with several size exemplars and testing them with novel exemplars that fall within the trained ones. In general, birds chose the relative flower more often when the test was upscaled rather than downscaled in size, a robust outcome that was not driven by variation between subjects (electronic supplementary material).

Whether larger is better will depend on the context. A larger resource might be better, but a larger adversary is worse than a smaller one. Depending on the context (e.g. foraging, choosing a refuge, or level of resource competition), relational rules might be useful when animals make quick choices. In the case of our hummingbirds, they learned that the larger or smaller flower could have both a larger or a smaller reward, but an intrinsic bias for larger flowers seemed also to occur.

Given the dynamic, changing nature of a hummingbird's territory, foraging presents a complex cognitive task. It is, therefore, worth considering that although conceptual learning has generally been considered a ‘higher’ cognitive process, it may be an essential tool for animals to forage successfully in such an environment. Research on honeybees, which face similar foraging challenges, has shown that bees too are able to use various relational concepts in a foraging context [44]. It is possible then that the use of relational rules is widespread and part of the cognitive abilities of many more animals than previously thought.

## Data Availability

All data are archived in the electronic supplementary material for this article [45].
