# Supplementary material for: Size is relative: use of relational concepts by wild hummingbirds
Source: Proc Biol Sci. 2022 Mar 23;289(1971):20212508. doi: 10.1098/rspb.2021.2508 (PMC8941385; doi:10.1098/rspb.2021.2508)
Supplement: Supplementary file 1 [file rspb20212508_review_history.pdf]

THE ROYAL SOCIETY
PUBLISHING

PROCEEDINGS B

Size is relative: use of relational concepts by wild hummingbirds

Theo Brown, T. Andrew Hurly, Susan D. Healy and Maria C. Tello-Ramos

Article citation details
Proc. R. Soc. B **289**: 20212508.
http://dx.doi.org/10.1098/rspb.2021.2508

Review timeline
Original submission: 16 November 2021
Revised submission: 26 January 2022
Final acceptance: 22 February 2022

Note: Reports are unedited and appear as submitted by the referee. The review history appears in chronological order.

Review History

RSPB-2021-2508.R0 (Original submission)

Review form: Reviewer 1

Recommendation
Major revision is needed (please make suggestions in comments)

Scientific importance: Is the manuscript an original and important contribution to its field?
Good

General interest: Is the paper of sufficient general interest?
Good

Quality of the paper: Is the overall quality of the paper suitable?
Good

Is the length of the paper justified?
Yes

Should the paper be seen by a specialist statistical reviewer?
No

Reports © 2022 The Reviewers; Decision Letters © 2022 The Reviewers and Editors; Responses © 2022 The Reviewers, Editors and Authors. Published by the Royal Society under the terms of the Creative Commons Attribution License http://creativecommons.org/licenses/by/4.0/, which permits unrestricted use, provided the original author and source are credited

Do you have any concerns about statistical analyses in this paper? If so, please specify them explicitly in your report.
No

It is a condition of publication that authors make their supporting data, code and materials available - either as supplementary material or hosted in an external repository. Please rate, if applicable, the supporting data on the following criteria.

Is it accessible?
N/A

Is it clear?
N/A

Is it adequate?
N/A

Do you have any ethical concerns with this paper?
No

Comments to the Author
The authors investigated the ability of wild hummingbirds to apply relational concepts in foraging contexts. The main goal was testing if wild male territorial rufous Hummingbirds are able to use "larger than" and "smaller than" relational rules and apply them to flowers of different sizes. Flowers contained the same amount of nectar but varied in quality.

The general concept investigated in this paper is interesting and relevant. The manuscript is well written and clearly describes the procedures and main findings of the experiments. The experimental set up also provides the appropriate controls for hummingbird cognitive tests, including replacement of flowers between visits and spatial location. However, I have a major comment. There is no clear explanation in this manuscript on how the experimental stimulus to test the application of relational concepts were chosen. There is abundant evidence (even cited by the authors Ref #22, Fenster et al. 2006) showing that variations in flower size is associated with variations in nectar quantity. On the contrary, there is no evidence of flower's size associated with variations in nectar quality (i.e., nectar concentration). Although I see the value of testing the ability of hummingbirds to apply a completely abstract concept, the problem could have been investigated in an evolutionary context if variations in size had been associated with variations in the quantity of nectar offered. Furthermore, between lines 85 – 91, the manuscript points to variations in nectar quantity, but there is no connection with the use of nectar quality in the methodology. Moreover, some of the inconsistent results ("down scale" tests did not differ from chance) could have been different in a more realistic scenario (lines 313-314).
Minor comments
- The methodology is well described and provides all the necessary information to follow the experimental procedures. Thus, Figure 1. is redundant (and confusing).
- The authors provide evidence against the alternative explanations for the results (peak shift and associative learning), even including this in Table 1. Thus, the manuscript will be clearer if a description of associative learning and peak shift were included in the introduction. They could be presented as alternative hypothesis.

Review form: Reviewer 2

Recommendation
Accept with minor revision (please list in comments)

Scientific importance: Is the manuscript an original and important contribution to its field?
Good

General interest: Is the paper of sufficient general interest?
Good

Quality of the paper: Is the overall quality of the paper suitable?
Good

Is the length of the paper justified?
Yes

Should the paper be seen by a specialist statistical reviewer?
No

Do you have any concerns about statistical analyses in this paper? If so, please specify them explicitly in your report.
No

It is a condition of publication that authors make their supporting data, code and materials available - either as supplementary material or hosted in an external repository. Please rate, if applicable, the supporting data on the following criteria.

> **Is it accessible?**
> Yes
>
> **Is it clear?**
> Yes
>
> **Is it adequate?**
> Yes

Do you have any ethical concerns with this paper?
No

Comments to the Author
I really enjoyed this article and I think the readers of Proceedings would like it, as well. The ms is well-written, gets right to the point, and the study is well-designed. I was all set to write that the authors should test a 'smaller than' discrimination after reading about the 'larger than' discrimination, but of course they had thought to wisely include that as I read on (as well as counterbalancing the order of training)! I also really like that ideas about relational learning are being tested by the authors with field work in this study.

My three substantive comments are:

1. I wonder, as do the authors in the discussion, about what might be causing the poorer discrimination of the small vs. tiny array combination (in particular). Is it some predisposition for large, as suggested by the initial acquisition data, or is it something to do with the physical differences in the arrays, or both? The absolute difference in surface area between the large and medium comparison and then the small vs. tiny stimuli is substantial, even if the ratios are more similar (though those still point to a potentially more challenging discrimination for the smallest stimuli). I was looking for evidence of this in the data set, but I think it is unclear if the birds can perceptually discriminate the small and tiny stimuli. I would have liked to have seem a bit more exploration of the later possibility, since this is the second most interesting finding from the study. For instance, what if a group of birds had more divergent initial training differences

(Large vs Tiny) and then the middle pairs were tested. Presumably there would be less time acquiring this discrimination than the medium-small training pairs and the birds may have had less problem with the small x tiny discrimination if they could perceive the size differences? I think not having addressed that further weakens the story somewhat. Perhaps that was a constrain of the field season?

2. Why did it take the birds so long to reacquire the discrimination after the initial test? If they had learned about the relations, would that rule have been so seriously disrupted by one unrewarded probe? Also, I note that there appears to be a learning effect that is worth mentioning (I don't think the reacquisition data was analyzed). That is, the number of trials it takes for the birds to reacquire the discrimination does go down between the first and second and then the 3rd and 4th periods of reacquisition (Figure 3) – a nice learning effect.

3. This is more of a minor point, but in the discussion the authors argue that they had not trained a relational rule. I think this is true compared to some of the work cited in the introduction. However, I would argue that they had trained a relational rule - just with one exemplar – again a minor point.

Decision letter (RSPB-2021-2508.R0)

06-Jan-2022

Dear Dr Tello-Ramos:

Your manuscript has now been peer reviewed and the reviews have been assessed by an Associate Editor. The reviewers' comments (not including confidential comments to the Editor) and the comments from the Associate Editor are included at the end of this email for your reference. As you will see, the reviewers and the Editors have raised some concerns with your manuscript and we would like to invite you to revise your manuscript to address them.

We do not allow multiple rounds of revision so we urge you to make every effort to fully address all of the comments at this stage. If deemed necessary by the Associate Editor, your manuscript will be sent back to one or more of the original reviewers for assessment. If the original reviewers are not available we may invite new reviewers. Please note that we cannot guarantee eventual acceptance of your manuscript at this stage.

To submit your revision please log into http://mc.manuscriptcentral.com/prsb and enter your Author Centre, where you will find your manuscript title listed under "Manuscripts with Decisions." Under "Actions", click on "Create a Revision". Your manuscript number has been appended to denote a revision.

When submitting your revision please upload a file under "Response to Referees" - in the "File Upload" section. This should document, point by point, how you have responded to the reviewers' and Editors' comments, and the adjustments you have made to the manuscript. We require a copy of the manuscript with revisions made since the previous version marked as 'tracked changes' to be included in the 'response to referees' document.

Your main manuscript should be submitted as a text file (doc, txt, rtf or tex), not a PDF. Your figures should be submitted as separate files and not included within the main manuscript file.

When revising your manuscript you should also ensure that it adheres to our editorial policies (https://royalsociety.org/journals/ethics-policies/). You should pay particular attention to the following:

Research ethics:
If your study contains research on humans please ensure that you detail in the methods section whether you obtained ethical approval from your local research ethics committee and gained informed consent to participate from each of the participants.

Use of animals and field studies:
If your study uses animals please include details in the methods section of any approval and licences given to carry out the study and include full details of how animal welfare standards were ensured. Field studies should be conducted in accordance with local legislation; please include details of the appropriate permission and licences that you obtained to carry out the field work.

Data accessibility and data citation:
It is a condition of publication that you make available the data and research materials supporting the results in the article. Please see our Data Sharing Policies (https://royalsociety.org/journals/authors/author-guidelines/#data). Datasets should be deposited in an appropriate publicly available repository and details of the associated accession number, link or DOI to the datasets must be included in the Data Accessibility section of the article (https://royalsociety.org/journals/ethics-policies/data-sharing-mining/). Reference(s) to datasets should also be included in the reference list of the article with DOIs (where available).

In order to ensure effective and robust dissemination and appropriate credit to authors the dataset(s) used should also be fully cited and listed in the references.

If you wish to submit your data to Dryad (http://datadryad.org/) and have not already done so you can submit your data via this link http://datadryad.org/submit?journalID=RSPB&manu=(Document not available), which will take you to your unique entry in the Dryad repository.

If you have already submitted your data to dryad you can make any necessary revisions to your dataset by following the above link.

For more information please see our open data policy http://royalsocietypublishing.org/data-sharing.

Electronic supplementary material:
All supplementary materials accompanying an accepted article will be treated as in their final form. They will be published alongside the paper on the journal website and posted on the online figshare repository. Files on figshare will be made available approximately one week before the accompanying article so that the supplementary material can be attributed a unique DOI. Please try to submit all supplementary material as a single file.

Online supplementary material will also carry the title and description provided during submission, so please ensure these are accurate and informative. Note that the Royal Society will not edit or typeset supplementary material and it will be hosted as provided. Please ensure that the supplementary material includes the paper details (authors, title, journal name, article DOI). Your article DOI will be 10.1098/rspb.[paper ID in form xxxx.xxxx e.g. 10.1098/rspb.2016.0049].

Please submit a copy of your revised paper within three weeks. If we do not hear from you within this time your manuscript will be rejected. If you are unable to meet this deadline please let us know as soon as possible, as we may be able to grant a short extension.

Thank you for submitting your manuscript to Proceedings B; we look forward to receiving your revision. If you have any questions at all, please do not hesitate to get in touch.

Best wishes,
Professor Gary Carvalho
mailto: proceedingsb@royalsociety.org

Associate Editor
Comments to Author:
We have now obtained two expert reviews of your manuscript, and I am happy to tell you that both the reviewers liked your paper. My own reading of your manuscript matches the reviews: this is an interesting piece of work that could become publishable in Proceedings B. However, there are still some minor revisions that need to be addressed expeditiously so I would appreciate if you could please deal with these in a revised version. These include the following additions suggested by the reviews. First, there should be more justification for use of variation in reward quality rather than quantity and some discussion of the potential shortcomings of this approach. Second, adding peak shift and associative learning as alternative hypotheses in the introduction would help the reader anticipate the results. Third, the Discussion should include more exploration of the issue of the ability to discriminate the Small and Tiny stimuli and how this might limit the findings. I see that the Medium Downscale test suggests they might be able to make this discrimination, but the sample sizes are small and inconclusive. It seems appropriate to acknowledge this as a potential shortcoming worthy of further exploration in the Discussion. Fourth, include additional discussion of Figure 2 and how long it took animals to reacquire the training (perhaps indicating which of the two criteria appeared to be limiting at this stage might help) and how Figure 2 indicates learning across sessions.

Reviewer(s)' Comments to Author:
Referee: 1
Comments to the Author(s)
The authors investigated the ability of wild hummingbirds to apply relational concepts in foraging contexts. The main goal was testing if wild male territorial rufous Hummingbirds are able to use “larger than” and “smaller than” relational rules and apply them to flowers of different sizes. Flowers contained the same amount of nectar but varied in quality.

The general concept investigated in this paper is interesting and relevant. The manuscript is well written and clearly describes the procedures and main findings of the experiments. The experimental set up also provides the appropriate controls for hummingbird cognitive tests, including replacement of flowers between visits and spatial location. However, I have a major comment. There is no clear explanation in this manuscript on how the experimental stimulus to test the application of relational concepts were chosen. There is abundant evidence (even cited by the authors Ref #22, Fenster et al. 2006) showing that variations in flower size is associated with variations in nectar quantity. On the contrary, there is no evidence of flower’s size associated with variations in nectar quality (i.e., nectar concentration). Although I see the value of testing the ability of hummingbirds to apply a completely abstract concept, the problem could have been investigated in an evolutionary context if variations in size had been associated with variations in the quantity of nectar offered. Furthermore, between lines 85 – 91, the manuscript points to variations in nectar quantity, but there is no connection with the use of nectar quality in the methodology. Moreover, some of the inconsistent results (“down scale” tests did not differ from chance) could have been different in a more realistic scenario (lines 313-314).
Minor comments
- The methodology is well described and provides all the necessary information to follow the experimental procedures. Thus, Figure 1. is redundant (and confusing).
- The authors provide evidence against the alternative explanations for the results (peak shift and associative learning), even including this in Table 1. Thus, the manuscript will be clearer if a description of associative learning and peak shift were included in the introduction. They could be presented as alternative hypothesis.

Referee: 2
Comments to the Author(s)
I really enjoyed this article and I think the readers of Proceedings would like it, as well. The ms is well-written, gets right to the point, and the study is well-designed. I was all set to write that the authors should test a 'smaller than' discrimination after reading about the 'larger than' discrimination, but of course they had thought to wisely include that as I read on (as well as counterbalancing the order of training)! I also really like that ideas about relational learning are being tested by the authors with field work in this study.

My three substantive comments are:

1. I wonder, as do the authors in the discussion, about what might be causing the poorer discrimination of the small vs. tiny array combination (in particular). Is it some predisposition for large, as suggested by the initial acquisition data, or is it something to do with the physical differences in the arrays, or both? The absolute difference in surface area between the large and medium comparison and then the small vs. tiny stimuli is substantial, even if the ratios are more similar (though those still point to a potentially more challenging discrimination for the smallest stimuli). I was looking for evidence of this in the data set, but I think it is unclear if the birds can perceptually discriminate the small and tiny stimuli. I would have liked to have seem a bit more exploration of the later possibility, since this is the second most interesting finding from the study. For instance, what if a group of birds had more divergent initial training differences (Large vs Tiny) and then the middle pairs were tested. Presumably there would be less time acquiring this discrimination than the medium-small training pairs and the birds may have had less problem with the small x tiny discrimination if they could perceive the size differences? I think not having addressed that further weakens the story somewhat. Perhaps that was a constrain of the field season?

2. Why did it take the birds so long to reacquire the discrimination after the initial test? If they had learned about the relations, would that rule have been so seriously disrupted by one unrewarded probe? Also, I note that there appears to be a learning effect that is worth mentioning (I don't think the reacquisition data was analyzed). That is, the number of trials it takes for the birds to reacquire the discrimination does go down between the first and second and then the 3rd and 4th periods of reacquisition (Figure 3) – a nice learning effect.

3. This is more of a minor point, but in the discussion the authors argue that they had not trained a relational rule. I think this is true compared to some of the work cited in the introduction. However, I would argue that they had trained a relational rule - just with one exemplar – again a minor point.

Author's Response to Decision Letter for (RSPB-2021-2508.R0)

See Appendix A.

RSPB-2021-2508.R1 (Revision)

Review form: Reviewer 1

Recommendation
Accept as is

Scientific importance: Is the manuscript an original and important contribution to its field?
Good

General interest: Is the paper of sufficient general interest?
Good

Quality of the paper: Is the overall quality of the paper suitable?
Good

Is the length of the paper justified?
Yes

Should the paper be seen by a specialist statistical reviewer?
No

Do you have any concerns about statistical analyses in this paper? If so, please specify them explicitly in your report.
No

It is a condition of publication that authors make their supporting data, code and materials available - either as supplementary material or hosted in an external repository. Please rate, if applicable, the supporting data on the following criteria.

> **Is it accessible?**
> Yes
>
> **Is it clear?**
> Yes
>
> **Is it adequate?**
> Yes

Do you have any ethical concerns with this paper?
No

Comments to the Author
The authors have adequately addressed all of my concerns in their revision and I believe the ms would make a good addition to the journal

Decision letter (RSPB-2021-2508.R1)

22-Feb-2022

Dear Dr Tello-Ramos

I am pleased to inform you that your manuscript entitled "Size is relative: use of relational concepts by wild hummingbirds" has been accepted for publication in Proceedings B.

You can expect to receive a proof of your article from our Production office in due course, please check your spam filter if you do not receive it. PLEASE NOTE: you will be given the exact page length of your paper which may be different from the estimation from Editorial and you may be asked to reduce your paper if it goes over the 10 page limit.

If you are likely to be away from e-mail contact please let us know. Due to rapid publication and an extremely tight schedule, if comments are not received, we may publish the paper as it stands.

If you have any queries regarding the production of your final article or the publication date please contact procb_proofs@royalsociety.org

Data Accessibility section
Please remember to make any data sets live prior to publication, and update any links as needed when you receive a proof to check. It is good practice to also add data sets to your reference list.

Open Access
You are invited to opt for Open Access, making your freely available to all as soon as it is ready for publication under a CCBY licence. Our article processing charge for Open Access is £1700. Corresponding authors from member institutions (http://royalsocietypublishing.org/site/librarians/allmembers.xhtml) receive a 25% discount to these charges. For more information please visit http://royalsocietypublishing.org/open-access.

Your article has been estimated as being 9 pages long. Our Production Office will be able to confirm the exact length at proof stage.

Paper charges
An e-mail request for payment of any related charges will be sent out after proof stage (within approximately 2-6 weeks). The preferred payment method is by credit card; however, other payment options are available

Electronic supplementary material:
All supplementary materials accompanying an accepted article will be treated as in their final form. They will be published alongside the paper on the journal website and posted on the online figshare repository. Files on figshare will be made available approximately one week before the accompanying article so that the supplementary material can be attributed a unique DOI.

Thank you for your fine contribution. On behalf of the Editors of the Proceedings B, we look forward to your continued contributions to the Journal.

Sincerely,
Professor Gary Carvalho
Editor, Proceedings B
mailto: proceedingsb@royalsociety.org

Appendix A

Response to referees

IN RE: RSPB-2021-2508

We thank the editor and reviewers for their comments and suggestions, which we believe have improved the manuscript. We have made all but one of the changes suggested (we have kept Figure 1). In the methods section, we have included our rationale for manipulating the quality of the reward rather than the quantity. We have also included more background on peak shift and associative learning as alternative mechanisms to relational learning. We have further discussed whether birds were able to discriminate between the Tiny and Small flowers and the number of trials taken to reacquire the task. Below we address each of the comments of the editor and reviewers (in bold letters).

Editor

We have now obtained two expert reviews of your manuscript, and I am happy to tell you that both the reviewers liked your paper. My own reading of your manuscript matches the reviews: this is an interesting piece of work that could become publishable in Proceedings B. However, there are still some minor revisions that need to be addressed expeditiously so I would appreciate if you could please deal with these in a revised version. These include the following additions suggested by the reviews.

First, there should be more justification for use of variation in reward quality rather than quantity and some discussion of the potential shortcomings of this approach.

In the methods section, we have included our justification for manipulating the concentration/ quality of the reward rather than the quantity. Briefly, our wild territorial hummingbirds do not fill their crops. Actually, they only fill their crops up to 10% during most foraging bouts and spend most of their time perching (70%). For these birds, during the breeding season, it seems more important that they carry less weight in order to chase off intruders and court females. In this scenario, a high concentration reward is more valuable than more reward. If we had manipulated quantity, discrimination between rewards would have been weaker.

Second, adding peak shift and associative learning as alternative hypotheses in the introduction would help the reader anticipate the results.

We have added peak shift and associative learning as alternative mechanisms at the end of the introduction.

Third, the Discussion should include more exploration of the issue of the ability to discriminate the Small and Tiny stimuli and how this might limit the findings. I see that the Medium Downscale test suggests they might be able to make this discrimination, but the sample sizes are small and inconclusive. It seems appropriate to acknowledge this as a potential shortcoming worthy of further exploration in the Discussion.

We have discussed further how the differences in flower size present during the two tests might have affected the results recorded. In short, we discuss how, given the training data, is clear that hummingbirds took longer to reach the criteria when the small flower was rewarded, which suggests a size bias towards bigger flowers. However, during the Medium downscale tests, when Small and Tiny were presented 11/16 birds did visit the correct relational flower. This suggests that most of the birds could perceive the difference between the two flowers. We discuss the shortcomings of this result and the methodology used. We suggest alternative manipulations that could have made this result clearer.

Fourth, include additional discussion of Figure 2 and how long it took animals to reacquire the training (perhaps indicating which of the two criteria appeared to be limiting at this stage might help) and how Figure 2 indicates learning across sessions.

We have discussed further the training data and the learning curve. We have mentioned that when the hummingbirds had to reverse learn the association between the previously unrewarded flower, birds took more trials to learn the new association. We suggest that this effect might be explained by proactive interference or by the interruption of the training protocol by the end of the day.

###Reviewer 1####

The authors investigated the ability of wild hummingbirds to apply relational concepts in foraging contexts. The main goal was testing if wild male territorial rufous
Hummingbirds are able to use “larger than” and “smaller than” relational rules and apply them to flowers of different sizes. Flowers contained the same amount of nectar but varied in quality.

The general concept investigated in this paper is interesting and relevant. The manuscript is well written and clearly describes the procedures and main findings of the experiments. The experimental set up also provides the appropriate controls

for hummingbird cognitive tests, including replacement of flowers between visits and spatial location. However, I have a major comment. There is no clear explanation in this manuscript on how the experimental stimulus to test the application of relational concepts were chosen. There is abundant evidence (even cited by the authors Ref #22, Fenster et al. 2006) showing that variations in flower size is associated with variations in nectar quantity. On the contrary, there is no evidence of flower's size associated with variations in nectar quality (i.e., nectar concentration). Although I see the value of testing the ability of hummingbirds to apply a completely abstract concept, the problem could have been investigated in an evolutionary context if variations in size had been associated with variations in the quantity of nectar offered.

Furthermore, between lines 85 – 91, the manuscript points to variations in nectar quantity, but there is no connection with the use of nectar quality in the methodology. Moreover, some of the inconsistent results ("down scale" tests did not differ from chance) could have been different in a more realistic scenario (lines 313-314).

We thank the reviewer for this comment. We have now included in the methods section our justification for manipulating the quality of the reward rather than the quantity. In short, given that wild territorial hummingbirds only fill their crops up to 10%. For these birds, during the breeding season, it is more important that they carry less weight in order to chase off intruders and to display to females. Thus, a high concentration reward is more valuable than more reward. If we had manipulated quantity, discrimination between the rewards would have been weaker.

Minor comments

- The methodology is well described and provides all the necessary information to follow the experimental procedures. Thus, Figure 1. is redundant (and confusing).

We believe the Figure 1 will help some readers understand the methodology with a single picture. We would like to keep it. We have included a sentence in the description of the figure to make it clearer.

- The authors provide evidence against the alternative explanations for the results (peak shift and associative learning), even including this in Table 1. Thus, the manuscript will be clearer if a description of associative learning and peak shift were included in the introduction. They could be presented as alternative hypothesis.

We have added peak shift and associative learning as alternative mechanisms at the end of the introduction, thank you for the suggestion.

###Reviewer 2###

I really enjoyed this article and I think the readers of Proceedings would like it, as well. The ms is well-written, gets right to the point, and the study is well-designed. I was all set to write that the authors should test a 'smaller than' discrimination after reading about the 'larger than' discrimination, but of course they had thought to wisely include that as I read on (as well as counterbalancing the order of training)! I also really like that ideas about relational learning are being tested by the authors with field work in this study.

My three substantive comments are:

1. I wonder, as do the authors in the discussion, about what might be causing the poorer discrimination of the small vs. tiny array combination (in particular). Is it some predisposition for large, as suggested by the initial acquisition data, or is it something to do with the physical differences in the arrays, or both? The absolute difference in surface area between the large and medium comparison and then the small vs. tiny stimuli is substantial, even if the ratios are more similar (though those still point to a potentially more challenging discrimination for the smallest stimuli). I was looking for evidence of this in the data set, but I think it is unclear if the birds can perceptually discriminate the small and tiny stimuli. I would have liked to have seem a bit more exploration of the later possibility, since this is the second most interesting finding from the study. For instance, what if a group of birds had more divergent initial training differences (Large vs Tiny) and then the middle pairs were tested. Presumably there would be less time acquiring this discrimination than the medium-small training pairs and the birds may have had less problem with the small x tiny discrimination if they could perceive the size differences? I think not having addressed that further weakens the story somewhat. Perhaps that was a constrain of the field season?

We agree that having presented more exemplars, including Large vs Tiny would have helped clarify the current results, as would a Medium vs Tiny comparison. At present, given the training data, is clear that hummingbirds took longer to reach the criteria when the small flower was rewarded, which suggests a size bias towards bigger flowers. During the Medium downscale tests, when Small and Tiny were presented 11/16 birds did visit the correct relational flower. This suggests that most of the birds could perceive the difference between the two flowers.

We do recognize the shortcomings of this unclear result and the methodology used. We have further discussed this finding and have suggested alternative manipulations that could have made this result clearer.

2. Why did it take the birds so long to reacquire the discrimination after the initial test? If they had learned about the relations, would that rule have been so seriously disrupted by one unrewarded probe? Also, I note that there appears to be a learning effect that is worth mentioning (I don't think the reacquisition data was analyzed). That is, the number of trials it takes for the birds to reacquire the discrimination does go down between the first and second and then the 3rd and 4th periods of reacquisition (Figure 3) – a nice learning effect.

We thank the reviewer for this comment. We think that birds took longer to reacquire the task due to two main reasons. First, as these experiments were done in the wild, sometimes, training was interrupted by the end of the day. This happened for the three birds that took the most trials to reach criteria during the third training set. Also, training took more trials when switching which flower was rewarded. This effect could be explained by proactive interference. We have discussed further the training data and the learning curve.

3. This is more of a minor point, but in the discussion the authors argue that they had not trained a relational rule. I think this is true compared to some of the work cited in the introduction. However, I would argue that they had trained a relational rule - just with one exemplar – again a minor point.

We argue that our protocol is different from training to use a relational rule. We did present an exemplar of two flowers that differ in size, but during training, birds were rewarded if they visited the flower with the correct size, so during training they could have been using associative learning. A truly relational rule training protocol includes multiple exemplars so that only the relational rule is associated with the reward. In our study, we did not do that. Instead, we just asked if spontaneously the birds that had received a reward in the large or small flower were using a relational rule, peak shift or indeed associative learning. We believe that hummingbirds can be trained to use a relational rule and in the future, we might train birds to do so before testing the application of such rules to novel contexts.

We have added a sentence explaining this point more clearly.
